# Emerging Dual-Gate FET Sensor Paradigm for Ultra-Low Concentration Cortisol Detection in Complex Bioenvironments

**DOI:** 10.3390/bios15030134

**Published:** 2025-02-22

**Authors:** Seung-Jin Lee, Won-Ju Cho

**Affiliations:** Department of Electronic Materials Engineering, Kwangwoon University, Gwangun-ro 20, Nowon-gu, Seoul 01897, Republic of Korea; seungjin98@kw.ac.kr

**Keywords:** field-effect transistor, dual-gate, extended-gate, capacitive coupling, cortisol sensor, antigen–antibody

## Abstract

Cortisol is a pivotal hormone regulating stress responses and is linked to various health conditions, making precise and continuous monitoring essential. Despite their non-invasive nature, conventional cortisol detection methods often suffer from inadequate sensitivity and reliability at low concentrations, limiting their diagnostic utility. To address these limitations, this study introduces a novel paradigm for high sensitivity cortisol detection using field-effect transistor (FET) sensors with dual-gate (DG) structures. The proposed sensor platform enhances sensitivity through capacitive coupling without requiring external circuits. Cortisol detection performance was evaluated by immobilizing monoclonal antibodies activated via 1-ethyl-3-(3-dimethylaminopropyl)carbodiimide and N-hydroxysuccinimide onto a SnO_2_ thin film-based extended-gate. The results revealed a sensitivity of 14.3 mV/dec in single-gate mode, which significantly increased to 243.8 mV/dec in DG mode, achieving a detection limit of 276 pM. Additionally, the reliability and stability of the sensor were validated by evaluating drift effects, confirming its ability to provide accurate detection even in artificial saliva environments containing interfering substances. In conclusion, the DG-FET-based cortisol detection approach developed in this study significantly outperforms conventional FET-based methods, enabling precise monitoring at ultra-low concentrations. This approach holds significant potential for diverse bioassays requiring high sensitivity and reliability in complex environments.

## 1. Introduction

Stress is a significant risk factor for various health conditions, including cardiovascular diseases, depression, and obesity [1]. It disrupts homeostasis by inducing prolonged immune suppression and inflammatory responses, leading to various illnesses that are difficult to treat and manage [2,3,4]. This highlights the importance of stress prevention and early diagnosis. Accurate methods for assessing stress levels are essential, with the detection of cortisol—a key biomarker of stress responses—receiving significant attention [5,6,7,8]. Cortisol, a critical hormone in stress regulation, is essential in the neuroendocrine system [9,10]. Secreted by the adrenal cortex, cortisol regulates energy metabolism and homeostasis. Current analytical methods for cortisol detection include enzyme-linked immunosorbent assays, Raman spectroscopy, chemiluminescent immunoassays, and chromatographic techniques [11,12,13,14]. While effective, these conventional methods often fail to achieve rapid detection, sensitivity, and accuracy, particularly in non-invasive applications [15,16,17,18]. Given that cortisol levels in human body fluids exhibit circadian fluctuations and are susceptible to behavioral and environmental factors, monitoring cortisol levels in readily accessible biofluids, such as sweat and saliva, is crucial for timely diagnosis and treatment [19,20,21]. In healthy adults, cortisol concentrations typically range from 8.16 to 141.7 ng/mL (22.5 nM–391.0 nM), while salivary cortisol levels are approximately two orders of magnitude lower, ranging from 0.1 to 10 ng/mL (276 pM–27.6 nM) [22,23,24,25]. These factors highlight the need for non-invasive, highly sensitive monitoring methods capable of detecting cortisol at low concentrations, driving the development of advanced cortisol sensors.

Ion-sensitive field-effect transistors (ISFETs), introduced by P. Bergveld in the early 1970s, have been widely adopted in physiological measurements and biosensing applications [26]. ISFETs offer distinct advantages, such as rapid response times and compatibility with complementary metal-oxide-semiconductor processes, making them suitable for mass production and miniaturization [27,28,29,30]. Despite these advantages, traditional ISFETs are limited by the Nernst limit (59.14 mV/pH at room temperature), which constrains their sensitivity, particularly for detecting low analyte concentrations such as biomolecules [31,32]. To overcome this limitation, dual-gate ISFETs (DG-ISFETs) have emerged as an effective alternative, offering enhanced sensitivity and stability [33,34,35]. The operating principles of ISFETs are governed by the site-binding and the Stern–Gouy–Chapman models, which describe the electrical double-layer theory of surface potential at the sensing membrane [36,37]. Interactions between ions in the electrolyte and surface binding sites of the sensing membrane result in changes in surface charge density, thereby modulating the surface potential in response to solution concentration. Recent advancements in ISFET technologies have expanded their application to detect diverse biomolecules, including DNA, cells, antigens, antibodies, and enzymes [38,39,40,41]. Many of these advancements rely on functionalized thin films to enhance detection sensitivity by altering surface charge density upon antigen–antibody binding [42]. Thin films that are highly sensitive to surface charge density changes, such as SnO_2_, are particularly advantageous for biosensing applications. SnO_2_ thin films are recognized for their high sensitivity, stability, rapid response, and cost efficiency, making them ideal materials for ISFET-based biosensors [43,44].

This study introduces a novel cortisol sensor that utilizes a dual-gate field-effect transistor (DG-FET) design. This sensor integrates a SnO_2_-based extended-gate (EG) functionalized with monoclonal antibodies specific to cortisol. The surface activation method ensures robust antibody immobilization, enhancing resistance to interference [45]. Additionally, the EG, positioned away from the active region, ensured consistent performance through its modular design, enabling attachment and detachment [46,47]. In the DG-FET, the transducer utilizes a top and bottom gate, forming two metal-oxide-semiconductor capacitor structures, enabling capacitive coupling amplification without external circuitry [48,49,50]. The fabricated DG-FET was systematically evaluated for its electrical properties, and the sensing performance of the SnO_2_ thin film was validated via pH-sensing. Atomic force microscopy (AFM) confirmed the effective immobilization of cortisol antibodies on the SnO_2_ surface. The detection performance was evaluated using cortisol solutions in phosphate-buffered saline (PBS) and artificial saliva. The DG-FET sensor demonstrated outstanding sensitivity, stability, and reliability at ultra-low concentrations, even amidst interfering substances. These results underscore its superior potential for stress monitoring compared to conventional FET-based cortisol sensors.

## 2. Materials and Methods

### 2.1. Materials

The materials used in this study included glass substrate (7059 glass; Corning Inc., Corning, NY, USA), deionized (DI) water with a conductivity of ≤4.3 μS/cm (Sigma-Aldrich, St. Louis, MO, USA), and Sylgard 184 polydimethylsiloxane (PDMS) elastomer (Dow Corning, Midland, MI, USA). Chemical reagents such as 1-ethyl-3-(3-dimethylaminopropyl)carbodiimide (EDC) (purity ≥ 97%, molecular weight = 155.24 g/mol; Sigma-Aldrich, USA) and N-hydroxysuccinimide (NHS) (purity ≥ 98%, molecular weight = 115.09 g/mol; Sigma-Aldrich, USA) were employed for surface functionalization. Other key materials included PBS at pH 7.4 (Sigma-Aldrich, USA), cortisol antibody (Clone BGN/C53, mouse monoclonal IgG3, concentration 1 mg/mL in PBS with 0.09% sodium azide; Thermo Fisher Scientific, (Waltham, MA, USA), cortisol solution (1.0 mg/mL in methanol, ampule of 1 mL; Sigma-Aldrich, USA), and artificial saliva (TB0929, 400 mL, pH 6.8, TMABio, Goyang, Republic of Korea).

### 2.2. Fabrication of DG-FET Transducer Unit and SnO_2_-Based EG-Sensing Membrane

**Figure 1:**
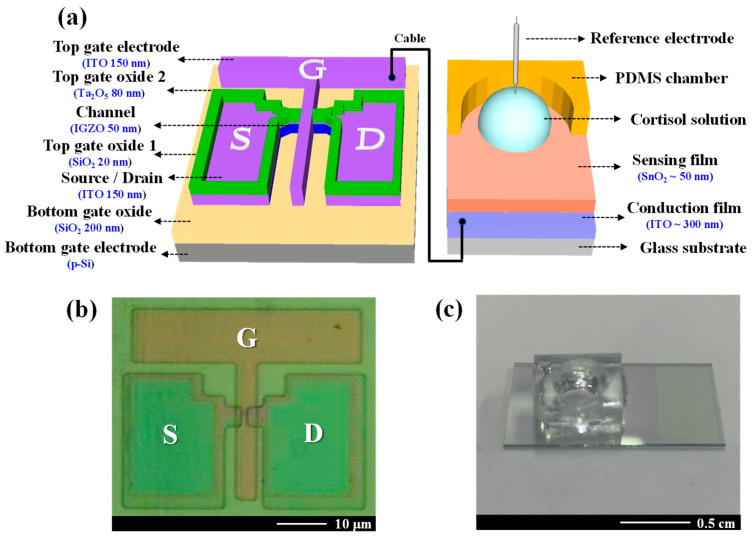
(**a**) A schematic diagram of the dual-gate field-effect transistor (DG-FET) architecture, highlighting the SnO_2_-based extended-gate (EG) sensing membrane. (**b**) The optical microscopy image of the fabricated DG-FET transducer unit shows the channel and electrode layout. (**c**) A photograph of the functionalized EG electrode functionalized with an immobilized cortisol antibody on the SnO_2_ surface.

The DG-FET for cortisol detection was fabricated using a sequential process. The bottom gate electrode was constructed using a p-type silicon (p-Si) substrate, which was cleaned using standard Radio Corporation of America cleaning protocols to remove surface impurities. A 200 nm thick SiO_2_ layer was deposited on the substrate as the bottom gate oxide using a radio frequency (RF) magnetron sputtering system. The active channel material was indium gallium zinc oxide (IGZO) with a stoichiometric ratio of In:Ga:Zn = 4:2:4.1, chosen for its high electron mobility [51,52]. The IGZO layer was initially deposited to a thickness of 50 nm using an RF magnetron sputtering system, then patterned into an active channel region measuring 20 μm in width and 10 μm in length through photolithography and wet etching techniques. A 150 nm thick indium-tin-oxide (ITO) layer was subsequently deposited to form the source and drain electrodes. The top gate oxide comprised a dual-layer structure to enhance the channel’s electrical properties. A 20 nm thick SiO_2_ layer acted as a buffer oxide, followed by an 80 nm thick tantalum oxide (Ta_2_O_5_) layer, which is a high-k dielectric material selected for its superior capacitance. The top gate electrode, consisting of a 150 nm thick ITO layer, was then deposited. To improve electrical properties and operational stability, forming gas annealing was conducted at 450 °C in a 5% H_2_/N_2_ atmosphere for 30 min. After annealing, the top gate electrode was connected to an EG-based electrode via a cable to enable full device functionality. The EG electrode incorporated a 300 nm thick ITO layer coated with a SnO_2_-sensing membrane. SnO_2_ was employed for its exceptional sensitivity and stability in detecting biomolecular interactions. Figure 1a,b presents the overall schematic and microscopy image of the fabricated DG-FET, respectively. Figure 1c shows the actual EG electrode with the immobilized cortisol antibody deposited onto the SnO_2_ surface.

### 2.3. Fabrication of Cortisol Antibody-Immobilized SnO_2_-Sensing Membrane in EG-Sensing Unit

**Figure 2:**
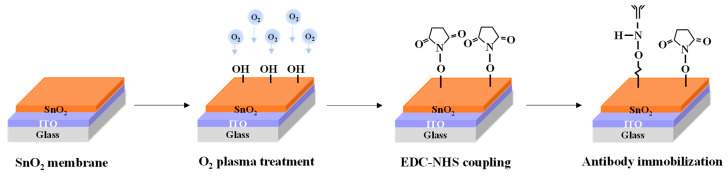
Schematic representation of fabrication process for immobilizing cortisol antibodies onto SnO_2_-sensing membranes in EG-sensing units.

An EG-sensing unit was fabricated using a 1.5 cm × 2.5 cm glass substrate. A 300 nm thick ITO conductive layer and a 50 nm thick SnO_2_-sensing layer were deposited sequentially using RF magnetron sputtering. Subsequently, a PDMS reservoir was installed to complete EG fabrication. To prepare the SnO_2_ surface for antibody immobilization, the fabricated EG underwent O_2_ plasma treatment for 30 s to generate hydroxyl (OH) groups [53,54]. The SnO_2_ surface was activated using a mixture of EDC and NHS, prepared by dissolving 0.479 g of EDC (molecular weight: 191.7 g/mol) and 0.288 g of NHS (molecular weight: 115.09 g/mol) in 50 mL of PBS to achieve a 50 mM solution. The EDC-activated carboxyl groups on the SnO_2_ surface, forming reactive O-acylisourea intermediates capable of binding to amine groups, while NHS stabilized these intermediates by converting them into more stable NHS esters, enhancing coupling efficiency [55,56]. A 1:1 mixture of EDC and NHS was applied to the SnO_2_ surface, with activation performed at room temperature for 30 min. The surface was then thoroughly rinsed with DI water. Following activation, the NHS esters reacted with the amine groups of the cortisol antibody, facilitating stable covalent immobilization [57,58]. A 10 μL droplet of a cortisol antibody solution (100 μg/mL in PBS buffer) was deposited onto the activated SnO_2_ surface and incubated overnight at 4 °C. After incubation, the EG surface was rinsed again with DI water to remove unbound antibodies. The process of fabricating the cortisol antibody-functionalized EG-sensing unit is shown in Figure 2.

### 2.4. DG-FET Operation Modes and Capacitive Coupling Effect

The fabricated DG-FET sensor operates in two distinct modes: single-gate (SG) and dual-gate (DG).

**Figure 3:**
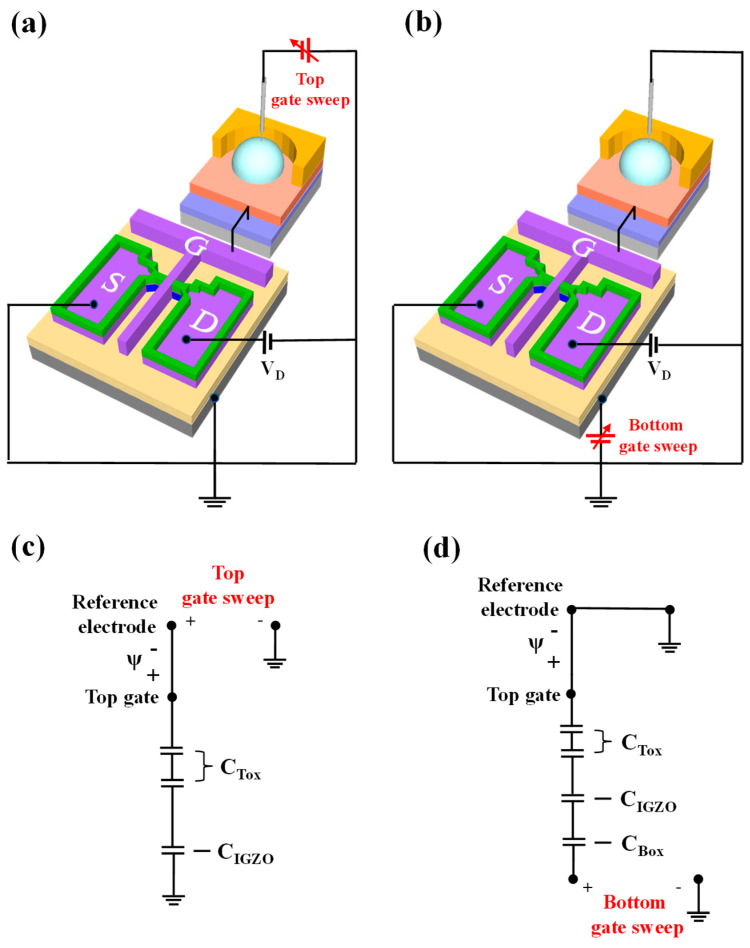
Schematic representation of DG-FET transducer unit in (**a**) single-gate (SG) and (**b**) dual-gate (DG) operation modes. Electrical equivalent circuits for (**c**) SG mode, showing threshold voltage relationship of top gate, and (**d**) DG mode, illustrating capacitive coupling effect between top and bottom gates and resulting self-amplification mechanism.

SG Mode:

In SG mode, only the top gate electrode is utilized, maintaining sensitivity and adhering to the Nernst limit characteristic of conventional ISFETs. A schematic of the SG mode operation is shown in Figure 3a. The equivalent circuit for SG mode is shown in Figure 3c. The threshold voltage of the top gate in SG mode is expressed as follows:

(1) $$\Delta V_{TH}^{TG}=-\Delta \psi$$

Dual-Gate (DG) Mode:

In the DG mode, both the top and bottom gate electrodes are utilized, resulting in self-amplified sensitivity due to the capacitive coupling effect between the two gates. A schematic of the DG mode operation is shown in Figure 3b, with the corresponding equivalent circuit depicted in Figure 3d. In the inversion region, the self-amplification effect is primarily determined by the capacitance ratio between the top gate insulator capacitance (*C_Tox_*) and the bottom gate insulator capacitance (*C_Box_*). For simplicity, the channel depletion capacitance (*C_IGZO_*) is neglected [48]. Therefore, the threshold voltage of the bottom gate, influenced by both the top and bottom capacitances, can be expressed as follows:

(2) $$\Delta V_{TH}^{BG}=\frac{C_{Tox}}{C_{Box}}\cdot \Delta V_{TH}^{TG}$$

Equation (2) illustrates that the threshold voltage of the bottom gate ($\Delta V_{TH}^{BG}$) is governed by the ratio *C_Tox_*/*C_Box_*, which defines the amplification ratio of DG-FET. To enhance the amplification ratio, the thickness of *C_Box_* can be increased, or materials with a lower dielectric constant can be used. For *C_Tox_*, a thinner layer or a high-dielectric constant film can be employed to improve amplification. In this study, a high-dielectric gate insulator structure, consisting of SiO_2_/Ta_2_O_5_ stacked oxide layers, was utilized to increase *C_Tox_*, resulting in a larger amplification ratio compared to a single SiO_2_ system. This dual-gate design significantly enhances the transducer’s performance, enabling a more precise detection of analyte concentrations through capacitive coupling.

### 2.5. Device Characteristics

The electrical characteristics of the DG-FET transducer, including its transfer and output properties, were measured using an Agilent 4156B precision semiconductor parameter analyzer (Agilent Technologies, Santa Clara, CA, USA). All measurements were conducted in an electromagnetically shielded dark box to minimize external interferences such as noise, light, and contamination. The sensing unit was connected to the transducer using RG58A 9222 electrical cables (BELDEN, St. Louis, MO, USA). Based on pH and cortisol concentrations, signal detection was performed by immersing the solutions in a PDMS reservoir, with a commercial Ag/AgCl electrode (2086A-06T, Horiba, Kyoto, Japan) employed as the reference electrode.

## 3. Results and Discussion

### 3.1. Electrical Characteristics of DG-FET

The electrical performance of the DG-FET transducer, particularly its sensitivity and reliability, was evaluated under two configurations involving the independent operation of the top and bottom gates. During these measurements, one gate electrode was actively biased while the other was grounded to isolate the behavior of each gate and investigate its contributions to the overall device performance.

**Figure 4:**
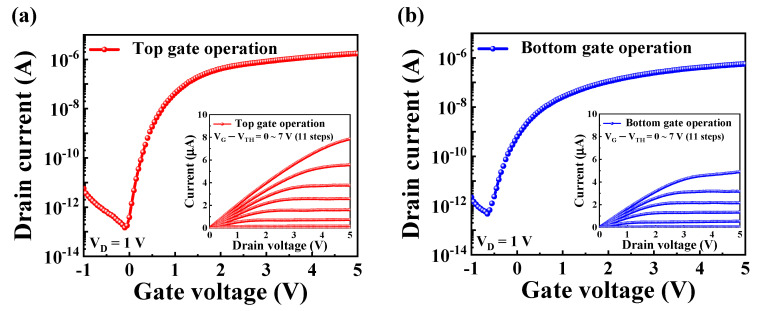
Transfer characteristic curves (*I*_*DS*_-*V*_*G*_) of DG-FET transducer during (**a**) top gate and (**b**) bottom gate operations. Inset images in each panel illustrate corresponding output characteristic curves (*I*_*DS*_-*V*_*D*_) obtained under varying gate voltages.

Figure 4a,b shows the transfer characteristics (*I*_*DS*_-*V*_*G*_) for the top and bottom gate operations, respectively. These curves were obtained by sweeping the gate voltage (*V*_*G*_) from −1 V to 5 V while maintaining the drain voltage (*V*_*D*_) at 1 V to ensure consistency in the measurement conditions. The transfer characteristics provide insights into the switching behavior and the linear and saturation regions of the operation transistor. The insets in each figure show the corresponding output characteristics, measured by varying the gate voltages from 0 V to 5 V. This setup enabled observation of the drain current (*I*_*DS*_) behavior under various operating conditions. During the output characteristic measurements, the overdrive voltage (*V*_*G*_−*V*_*TH*_) was systematically adjusted from 0 V to 7 V in 11 incremental steps. This systematic variation enabled a precise assessment of the transistor’s dynamic response and ability to modulate current flow.

Key electrical parameters of the DG-FET transducer, including threshold voltage (*V*_*TH*_), ON/OFF current ratio (*I*_*ON*_/*I*_*OFF*_), field-effect mobility (*μ*_*FE*_), and subthreshold swing (*SS*), were extracted from the *I*_*DS*_-*V*_*G*_ curves and are summarized in Table 1. For top gate operation, the device exhibited a *V*_*TH*_ of 0.25 V, an *I*_*ON*_/*I*_*OFF*_ of 1.18 × 10^7^, a *μ*_*FE*_ of 336.3 cm^2^/V·s, and a *SS* of 124.21 mV/dec, highlighting high performance and efficient current modulation. In comparison, the bottom gate operation exhibited a *V*_*TH*_ of −0.68 V, an *I*_*ON*_/*I*_*OFF*_ of 1.24 × 10^6^, a *μ*_*FE*_ of 107.7 cm^2^/V·s, and a *SS* of 184.39 mV/dec. The differences in electrical parameters between the top and bottom gates reflect their distinct contributions to the overall functionality of the DG-FET transducer. The higher ON/OFF current ratio and mobility observed for the top gate operation can be attributed to the optimized dielectric stack of SiO_2_/Ta_2_O_5_, which enhances the capacitance and overall control of the channel. This comparative analysis highlights the potential of the DG-FET transducer as a versatile, high-performance platform for sensing applications.

### 3.2. pH-Sensing Characteristics of the SnO_2_-Sensing Membrane-Based DG-FET

The pH-sensing capabilities of the DG-FET sensor, incorporating a SnO_2_ thin film as the sensing membrane, were investigated to assess its sensitivity, stability, and operational reliability. The sensing mechanism relies on the SnO_2_ film’s ability to modulate surface charge density in response to variations in hydrogen ion concentration, which alters the device’s electrical properties. The reference voltage (Δ*V*REF) measurements were conducted at a reference drain current (I_R_) of 100 pA, a condition optimized for maintaining consistent and reliable sensitivity.

**Figure 5:**
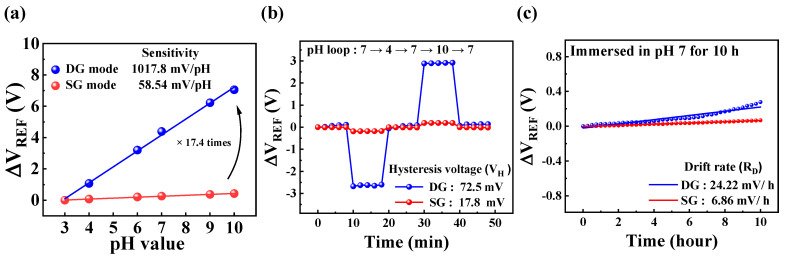
pH-sensing characteristics of DG-FET in SG and DG modes extracted from *I*_*DS*_-*V*_*G*_ curves: (**a**) Δ*V*_REF_ shift in response to pH values ranging from 3 to 10; (**b**) hysteresis effect observed during pH cycling through values 4, 7, and 10; and (**c**) drift behavior of sensing membrane immersed in pH 7 solution over 10 h.

Sensitivity Analysis:

Figure 5a illustrates the Δ*V*_REF_ response to pH variations across a range of 3 to 10. In SG mode, the SnO_2_-based DG-FET exhibited a pH sensitivity of 58.54 mV/pH, which is close to the theoretical Nernst limit. In contrast, the DG mode demonstrated an amplified sensitivity of 1017.8 mV/pH, approximately 17.4 times higher than the SG mode. This significant enhancement in sensitivity highlights the role of capacitive coupling between the top and bottom gates in amplifying the transducer’s response.

Hysteresis Analysis:

Figure 5b illustrates the voltage hysteresis (V_H_) behavior when pH was cycled through the following loop: pH 7 → pH 4 → pH 7 → pH 10 → pH 7. V_H_ was determined by calculating the difference between the initial and final V_REF_ values. The hysteresis width was found to be 17.8 mV in SG mode and 72.5 mV in DG mode. The hysteresis is attributed to the interaction dynamics of hydrogen ions with the SnO_2_ surface. Hydroxy groups on the sensing membrane surface exhibit rapid hydrogen ion capture or release, while ions diffusing into the bulk layer exhibit slower kinetics. This kinetic disparity leads to a lagged response during pH cycling, inducing hysteresis. To evaluate the reliability, the measurements were conducted over 50 min, with each pH solution equilibrated for 2–10 min.

Drift Behavior:

Figure 5c depicts the Δ*V*_REF_ drift over time, monitored using pH solutions stored in the PDMS reservoir. The drift is primarily caused by the formation of a hydration layer on the SnO_2_-sensing film, which facilitates ion diffusion into the sensing membrane, thereby altering its electrical properties. This gradual shift in Δ*V*_REF_ is represented as a time-dependent curve, with a drift rate (R_D_) of 6.86 mV/h in SG mode and 24.22 mV/h in DG mode. Despite the amplification of sensitivity in the DG mode, both V_H_ and R_D_ exhibited only modest increases, demonstrating the robustness of the SnO_2_ film.

In conclusion, these findings confirm the excellent sensitivity and reliable performance of the SnO_2_-sensing membrane for pH detection. While the DG mode significantly amplifies sensitivity, the slight increases in hysteresis and drift rates indicate that the SnO_2_ thin film maintains high stability and reliability under dynamic conditions. This validates the effectiveness of the DG-FET amplification mechanism of the transducer and highlights its suitability for pH-sensing applications, where precision and stability are critical. Additionally, it highlights the potential for adaptation to a wide range of analyte concentrations and varying environmental conditions.

### 3.3. AFM Analysis

**Figure 6:**
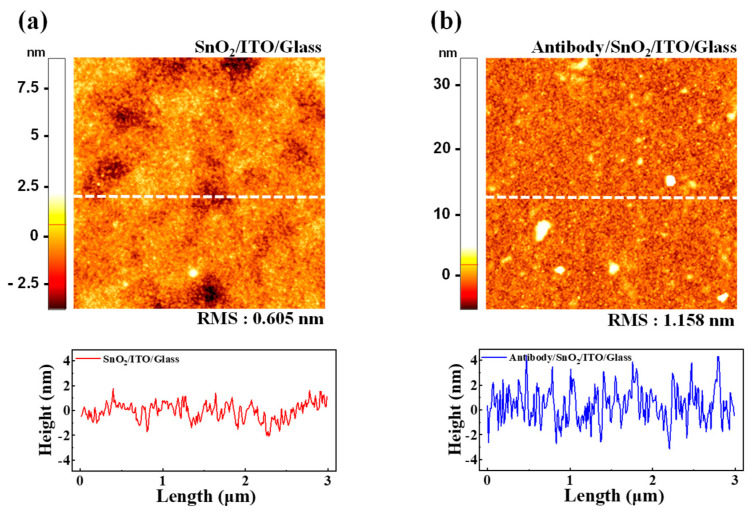
AFM images and corresponding line height profiles of SnO_2_-sensing membrane surface: (**a**) pristine SnO_2_/ITO/glass surface with RMS roughness of 0.605 nm, indicating smooth and flat surface. (**b**) Surface after immobilization of cortisol antibody, showing uniform biomolecular layer with increased RMS roughness of 1.158 nm. Measured height difference of approximately 0.553 nm confirms successful biofunctionalization for antigen binding.

AFM was employed to investigate surface morphological changes in the SnO_2_ thin film upon the immobilization of the cortisol antibody. Imaging was conducted using the SPM Solver-Pro system (NT-MDT, Zelenograd, Russia) in tapping mode, which enabled high-resolution, non-invasive surface topography analysis. The scanning area was set to 3 μm × 3 μm to ensure detailed visualization of the surface changes before and after antibody attachment. The AFM image of the pristine SnO_2_/ITO/glass surface is shown in Figure 6a. The topographical map revealed a smooth, uniform surface with a root mean square (RMS) roughness of 0.605 nm. A height profile of the marked section further corroborates the observation of a flat and homogeneous surface, confirming the suitability of the SnO_2_ thin film for subsequent biofunctionalization. Figure 6b illustrates the AFM image of the same surface following the immobilization of the cortisol antibody. The entire surface appeared densely covered, indicating successful antibody attachment. The RMS roughness increased to 1.158 nm, reflecting an additional layer formed by the immobilized biomolecules. The height difference of approximately 0.553 nm between the pristine and functionalized surfaces aligns with previously reported molecular dimensions of the cortisol antibody [59,60,61]. These findings confirm the effective biofunctionalization of the SnO_2_ thin film, which creates a robust interface for specific cortisol antigen interactions. Such surface modifications are critical for ensuring high sensitivity and selectivity in biosensing applications, as the uniform immobilization of biomolecules directly affects the transduction efficiency of the sensing platform.

### 3.4. Sensing Performance of DG-FET for Cortisol Detection

After validating the operational characteristics of the fabricated DG-FET, the SnO_2_-based EG-sensing membrane, and the surface biofunctionalization, the performance of the sensor for cortisol detection was evaluated. The sensing characteristics were tested in both PBS and artificial saliva environments, with sensitivity measured by shifts in the Δ*V*_REF_ at a constant I_R_ of 10 pA, corresponding to variations in cortisol concentration.

**Figure 7:**
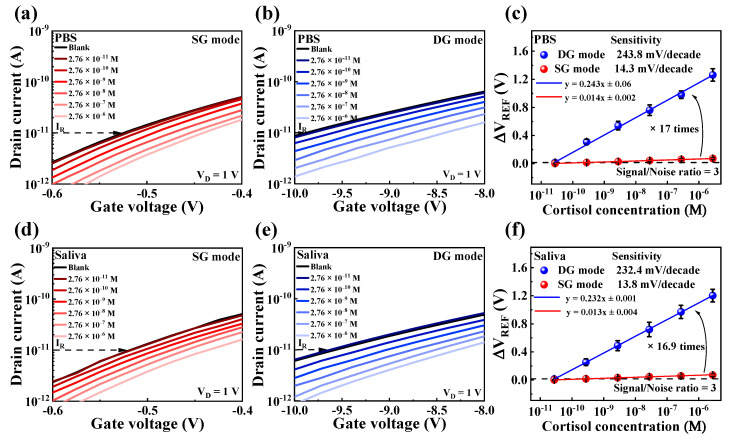
*I*_*DS*_-*V*_*G*_ transfer curves of DG-FET sensor under varying cortisol concentrations in (**a**) SG mode and (**b**) DG mode in PBS buffer solution, and in (**d**) SG mode and (**e**) DG mode in artificial saliva. Sensitivity comparisons and signal-to-noise ratio (S/N = 3) in (**c**) PBS buffer solution and (**f**) artificial saliva illustrate amplification achieved in DG mode compared to SG mode. Error bars show standard deviation for three different DG-FET sensors.

Figure 7a,b shows the *I*_*DS*_-*V*_*G*_ curves of DG-FET in the SG and DG modes, respectively, under varying cortisol concentrations in PBS. As cortisol concentration increased, the transfer characteristic curves shifted progressively in the positive V_G_ direction, demonstrating effective detection.

A comparative analysis summarized in Figure 7c reveals that the sensitivity in DG mode was approximately 17 times higher than in SG mode. This significant amplification is attributed to the capacitive coupling effect inherent in the dual-gate configuration.

Figure 7d,e shows the transfer characteristic curves in SG and DG modes, respectively, when tested in artificial saliva. A similar trend of positive shifts in transfer curves with increasing cortisol concentrations was observed. Figure 7f shows that the sensitivity in the DG mode was amplified by approximately 16.9 times compared to the SG mode, corroborating the findings in PBS.

As summarized in Table 2, these results confirm that while the DG-FET sensor exhibits a comparable limit of detection (LOD) to other recent FET-based cortisol sensors, it demonstrates superior sensitivity at low concentrations.

The limit of detection of the sensor was calculated using the formula LOD = 3⋅RMS_noise_/*m*, where RMS_noise_ represents the root mean square noise, and *m* represents the slope from the linear fitting of the sensor’s response (*y* = *mx* + *c*) [62,63]. The RMS noise was calculated based on repeated blank measurements (10 replicates) conducted under identical conditions in both SG and DG modes, for both PBS and artificial saliva, without cortisol. The standard deviations were 4.8 mV and 92 mV for the SG and DG modes in PBS, and 5.4 mV and 83 mV for the SG and DG modes in artificial saliva, respectively. Based on these measurements, the LOD values were determined to be 246 pM, 262 pM, 273 pM, and 268 pM. The LOD for the proposed cortisol sensor was defined as 276 pM, based on the measured concentrations. As is consistent with previous studies, the DG configuration did not significantly improve the LOD [64].

Nevertheless, the DG-FET sensor demonstrated the potential for highly sensitive cortisol detection at nanomolar concentrations, making it suitable for biosensing applications in complex biofluids such as artificial saliva.

**Table 2:** Performance comparison with recent FET-based cortisol sensors.

Platform	Sample	Sensitivity	LOD	Ref.
Aptamer/Graphene/Pt	Sweat	11.9~14.7 mV/decade	0.2 nM	[65]
Aptamer/Graphene	PBS	3.34 mV/dec	1 nM	[66]
Anticortisol/PSMA/ITO	PBS	10 fg/mL	10 ng/mL	[67]
Aptamer/SiNW/Ag	Saliva	74.7 mV/dec	0.005 μg/dL	[68]
Antibody/SnO_2_/ITO	Saliva	232.4 mV/decade	0.27 nM	This work

**Figure 8:**
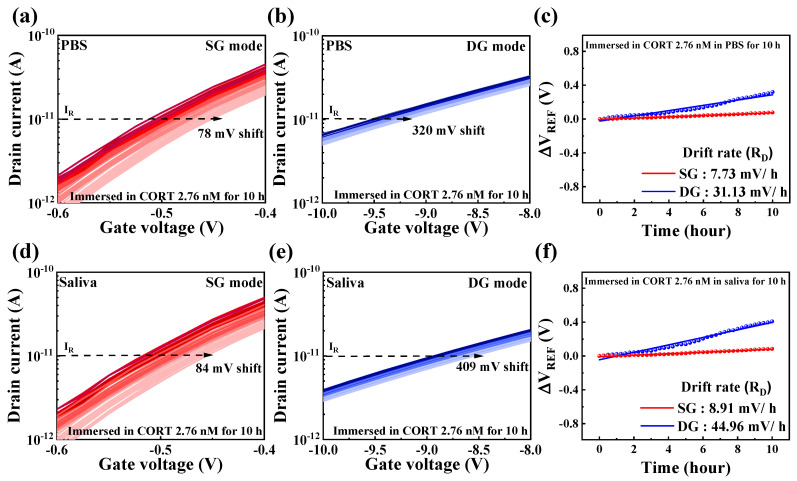
Drift effects were observed at cortisol concentration of 2.76 nM in (**a**) SG mode and (**b**) DG mode within PBS buffer solution and in (**d**) SG mode and (**e**) DG mode within artificial saliva. Drift rate, calculated based on shift in Δ*V*_REF_ over time, is shown in (**c**) for PBS buffer solution and (**f**) for artificial saliva. These results highlight stability and reliability of DG-FET transducer under extended operation, with DG mode demonstrating higher drift rates due to amplified sensitivity.

To evaluate the reliability and stability of the cortisol sensor, drift effects were systematically analyzed over prolonged periods under conditions simulating human saliva concentrations (2.76 nM cortisol). The drift was quantified by measuring the difference between the initial and final Δ*V*_REF_ values. As is consistent with the methodology employed for sensitivity measurements, the voltage sweep was reduced to 1/10 to minimize noise, and changes in Δ*V*_REF_ at 10 pA were monitored. Figure 8a,b shows the repeatedly measured *I*_*DS*_-*V*_*G*_ transfer curves of DG-FET in PBS buffer solution for the SG and DG modes, respectively. In SG mode, the transfer curves exhibited a positive drift of 78 mV over the testing period, whereas in DG mode, the shift was more significant at 320 mV. Figure 8c summarizes the drift rates calculated from these repeated measurements, with values of 7.73 mV/h in SG mode and 31.13 mV/h in DG mode. Similarly, Figure 8d,e shows the repeatedly measured *I*_*DS*_-*V*_*G*_ transfer curves for the DG-FET sensor in artificial saliva. A positive shift of 84 mV was observed in SG mode, whereas DG mode exhibited a larger shift of 409 mV. The corresponding drift rates, summarized in Figure 8f, were calculated as 8.91 mV/h for SG mode and 44.96 mV/h for DG mode. These results demonstrate that the drift rates in the DG mode were approximately four to five times higher than those in the SG mode for both PBS and artificial saliva environments. This behavior underscores the trade-off between the enhanced sensitivity of the DG mode and its reduced drift stability. Nevertheless, the SnO_2_-based extended-gate (EG) structure and the DG-FET transducer exhibited reliable and stable performance across extended testing periods, validating their suitability for sensitive cortisol detection even under dynamic and physiological conditions.

### 3.5. Quantitative Comparison of Amplification and Drift in Cortisol Sensing Using DG-FET

**Figure 9:**
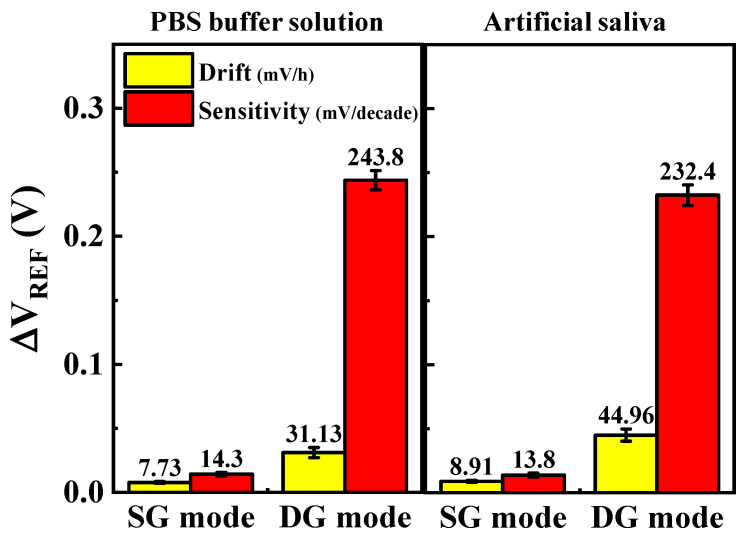
Quantitative comparison of sensitivity to cortisol concentrations (0–2.76 μM) and drift effects over 10 h in 2.76 nM cortisol solution. Sensitivity and drift rates are shown for SG and DG modes in PBS buffer and artificial saliva. Error bars show standard deviation for three different DG-FET sensors. Results highlight significant amplification in sensitivity achieved with DG mode despite increased drift effects, demonstrating sensor’s high sensitivity and reliability in both environments.

Figure 9 presents a quantitative analysis comparing the change in V_REF_ with the overall cortisol concentration (0–2.76 μM), derived from the sensitivity measurements in Figure 7, and the change in V_REF_ over 10 h at a single concentration (2.76 nM), obtained from the drift measurements in Figure 8. V_REF_ changes were more significant in the DG mode than in the SG mode, as evidenced by the increased drift rates. However, the significant enhancement in sensitivity achieved through the amplification effect in the DG mode outweighed this limitation, rendering the drift difference negligible in practical applications, as confirmed by the V_REF_ changes.

The comparison of V_REF_ magnitudes based on sensitivity and drift in PBS and artificial saliva environments revealed consistent results, demonstrating the sensor’s robustness against interference. The use of monoclonal cortisol antibodies on the SnO_2_-based sensing membrane proved to be highly effective in ensuring reliable and specific cortisol detection. Furthermore, integrating the dual-gate amplification mechanism enhanced sensitivity and mitigated the adverse impact of drift effects.

Therefore, in real-time detection environments, drift mitigation resulting from high sensitivity ensures the stable and precise detection of concentration changes even during prolonged repeated measurements, further enhancing the applicability of DG mode. In the future, introducing a nanostructured SnO_2_ film, which increases the surface area-to-volume ratio, is expected to further enhance sensitivity while effectively reducing drift.

These findings highlight the exceptional potential of the proposed DG-FET-based cortisol sensor. By combining high sensitivity with stability across diverse operational environments, the sensor presents significant advantages for various biosensing applications. Its ability to detect low cortisol concentrations with minimal interference or instability highlights its suitability for integration into advanced diagnostic tools and broader biosensing platforms.

## 4. Conclusions

This study presents a novel DG-FET-based sensor paradigm designed for the ultra-low concentration detection of cortisol in complex bioenvironments. Cortisol, a key biomarker for stress and health-related conditions, requires highly sensitive and reliable detection methods, particularly in physiologically relevant environments such as PBS buffer and artificial saliva. The proposed DG-FET sensor platform demonstrated significant advances in both sensitivity and stability by leveraging the amplification effect inherent in the dual-gate structure. Electrical characterization revealed an approximately 17-fold increase in sensitivity in DG mode compared to operations mode, highlighting the amplification potential for detecting subtle variations in surface charge density. Functionalization of the SnO_2_-sensing membrane was confirmed via AFM analysis, verifying its capability for selective cortisol binding and precise detection even in complex biofluids. Detection limits of 246 pM in PBS buffer and 273 pM in artificial saliva were achieved, highlighting the sensor’s exceptional performance in both environments. Although drift effects were observed, particularly in DG mode, the amplification provided by the dual-gate configuration more than compensated for this limitation, ensuring high reliability and minimal interference during repeated measurements at the same concentration over time. Furthermore, the demonstrated stable performance over extended periods confirmed its practical applicability for real-world biosensing scenarios. The originality of this work lies in integrating dual-gate FET technology with SnO_2_-based sensing membranes, which significantly enhances sensitivity and stability for cortisol detection. This innovative approach addresses the limitations of conventional biosensors and offers a scalable and adaptable platform for future sensing applications. The findings underscore the necessity of advanced sensor technologies like DG-FET to meet the growing demand for ultra-sensitive, stable, and reliable biosensors in complex environments. In conclusion, this study proposes a transformative DG-FET-based cortisol sensor paradigm for ultra-low concentration detection. The sensor holds considerable promise for various applications, including clinical diagnostics, stress monitoring, and environmental health assessments, providing a robust foundation for next-generation biosensing technologies.

## Figures and Tables

**Table 1:** A summary of the electrical parameters of the DG-FET transducer unit for different gate operation modes.

Operation Mode	*V*_*TH*_ (V)	*I*_*ON*_/*I*_*OFF*_ (A/A)	*μ*_*FE*_ (cm^2^/V·s)	*SS* (mV/dec)
Top Gate	0.25	1.18 × 10^7^	336.3	124.21
Bottom Gate	−0.68	1.24 × 10^6^	107.7	184.39

## Data Availability

Data are contained within the article.
